# Understanding the clinical and molecular basis of thyroid orbitopathy: a review of recent evidence

**DOI:** 10.1007/s42000-023-00498-8

**Published:** 2023-11-01

**Authors:** Pratheeba Devi Nivean, Nivean Madhivanan, Govindasamy Kumaramanikavel, Tos T. J. M. Berendschot, Carroll A. B. Webers, Dion Paridaens

**Affiliations:** 1 M.N Eye Hospital, Chennai, India; 2 Maastricht University, Maastricht, The Netherlands; 3 Genvams Trust, Chennai, India; 4 University Eye Clinic Maastricht, Maastricht, The Netherlands; 5 Department of Ophthalmology, Erasmus Medical Centre, Rotterdam, The Netherlands; 6 Orbital Service, Rotterdam Eye Hospital, Rotterdam, The Netherlands

**Keywords:** Thyroid eye disease, TED, Biomarkers, TED review, Thyroid orbitopathy, Graves’ disease, VISA grading, EUGOGO guidelines

## Abstract

Thyroid eye disease (TED) is an autoimmune orbital inflammatory disease which ranges from mild to severe. Tissue remodeling, fibrosis and fat proliferation cause changes in the orbital tissues which can affect esthetics and visual function. In its severe form, it is sight threatening, debilitating, and disfiguring and may lead to social stigma, the embarrassment about which has an impact on the quality of life of those affected and the family members. The pathogenesis of TED, which is influenced by genetic, immunological, and environmental factors, is complex and not fully elucidated. However, it remains unknown what factors determine the severity of the disease. Recent research has revealed a number of diagnostic and prognostic biomarkers of this disease. In this overview of TED, we focus on new insights and perspectives regarding biological agents that may provide a basis for new treatment modalities.

## Introduction

Autoimmune thyroid diseases (AITDs) are the most prevalent organ-specific autoimmune disorders [1]. They have a gender predilection, being more common among women than men [2]. AITDs include Graves’ disease (GD), resulting in hyperthyroidism, and Hashimoto’s disease (HD), which generally leads to hypothyroidism. They both induce an immunological imbalance that causes a cell-mediated autoantibody immune response against antigens of the thyroid gland [3]. The lymphocytic infiltration due to this immune imbalance leads to tissue damage to the thyroid gland, progressing to dysfunction. In HD, there is a predominance of lymphocytic infiltration of the thyroid gland, whereas in GD, there is autoantibody production against thyroid antigens.

### What is thyroid eye disease?

The eye symptoms and signs that occur associated with thyroid dysfunction are called thyroid eye disease (TED), also known as Graves’ ophthalmopathy or orbitopathy (GO), thyroid-associated ophthalmopathy (TAO), and thyroid orbitopathy (TAO). According to the literature, 90% of patients with TED have hyperthyroidism, 5% are hypothyroid, and another 5% are euthyroid, thus confirming that thyroid levels are not an isolated risk factor [4]. TED occurs in only 25–50% of patients with AITD [5]. Tanda et al. reported that 73.7% of newly diagnosed thyroid patients had no ocular involvement, 20.2% had mild and inactive TED, 5.8% had moderate to severe and active TED, and 0.3% had compressive optic neuropathy in an Italian population [6].

Lid retraction as a sign of TED was described in the nineteenth century by Dalrymple working at the Royal London Ophthalmic Hospital. The sign referred to as lid lag (also known as Graefe’s sign, named a/er the German ophthalmologist Von Graefe). Gorman and Bartley’s diagnostic criteria for TED are as follows. If eyelid retraction occurs in association with any one of the following: thyroid dysfunction, exophthalmos, optic nerve dysfunction, or extraocular muscle involvement, then the patient is said to have TED. If lid retraction is absent, then TED may be diagnosed only if exophthalmos, optic nerve involvement, or restrictive extraocular myopathy is associated with thyroid dysfunction [7].

Frueh’s diagnostic criteria are more objective for the diagnosis of TED [8]. Here, at least two of the following clinical features need to be met: a history of thyroid dysfunction, exophthalmos >20 mm, lid retraction (>7mm), extraocular muscle involvement, bilaterality on CT scan, or bilaterality of the above clinical features was proposed as being diagnostic of thyroid eye disease.

The natural course of TED has been described as following Rundle’s curve, which has two phases, namely, an active and an inactive phase [5]. The initial active inflammatory phase is characterized by periorbital erythema and edema, orbital inflammation, conjunctival chemosis and congestion, and upper lid retraction. The inflammatory phase typically lasts for a period of between 6 and 24 months and is followed by a quiet, minimally inflammatory chronic fibrotic phase, which is associated with orbital fibrosis, glycosaminoglycan deposition, and enlarged extraocular muscles.

## Epidemiology

TED is more common in females, with a female-to-male ratio that varies among studies and ranges from 1:4 to 8.01 [9]. In Europe, the prevalence of TED of all degrees is thought to be between 90 and 155/100,000 population and was estimated to be significantly higher in Caucasians than in Asians (42 vs. 7.7%, *p* = 0.0002), with the risk of developing TED 6.4-fold higher in Caucasians, this being attributed to the high prevalence of smoking (>60%) among Caucasians [10]. A recent meta-analysis, however, showed a slightly lower prevalence of TED among Caucasians (37%), compared to Asians (45%) [11]. Another meta-analysis reports the global prevalence of types of thyroid dysfunction as the following: in patients with thyroid eye disease, 10.36% had hypothyroidism, 7.9% were euthyroid, and 86.2% had hyperthyroidism. The existing literature reveals that TED is common in hyperthyroidism [12].

### Activity and severity of TED

Douglas et al. developed a clinical response index for TED using the Delphi method [13]. Their goal was to develop an instrument that can be used for clinical assessment in prospective, longitudinal, and observational clinical trials. Activity is measured by various scoring systems, such as the clinical activity score of Mourits et al., NO SPECS (no physical signs, only signs, soft tissue involvement, proptosis, extra ocular muscle involvement, corneal involvement, and sight loss due to optic nerve compression), and VISA (vision, inflammation, strabismus, and appearance) scoring [14]. VISA scoring is considered to be practical as it can prognosticate during follow-up visits. Based on the VISA classification, the disease is classified as active or inactive, where a score above 4 out of 8 is considered active. Active disease needs immediate treatment to avoid potential complications, whereas inactive disease can be managed conservatively [15]. Severity is assessed by the European Group on Graves’ orbitopathy (EUGOGO) [16]. Based on these guidelines, the disease is classified as mild, moderate, or severe. Thyroid-stimulating immunoglobulin can be detected by bioassays which serve as prognostic indicators of the activity of the disease with 97% sensitivity and 90% specificity.

### Effect of TED on quality of life

There is abundant literature evidence that TED affects quality of life (QOL). Terwee et al. developed a QOL questionnaire specific for TED with two subscales of eight questions each [17]. The first subscale was based on vision, including double vision, and the second on esthetic appearance. Park et al. modified the questionnaire by dividing it into three sections containing 19 questions [18]. According to several studies, TED dramatically decreases QOL. Delampady et al. validated TED QOL in the Hindi language in India and showed a reduction in QOL among those with TED [19]. They also added that in their population, there are various limitations in the visual function subscale in carrying out such activities as using public transport or working in agricultural fields, driving, and reading as most of their patients are from a rural background. Lee et al. [20] performed an elaborate analysis of existing questionnaires and reported that the TED questionnaire for QOL by Terwee et al. is faster and can be done in a busy outpatient department, whereas the Park et al. QOL questionnaire is elaborate and time-consuming. Stephanie et al. performed a meta-analysis to study QOL in TED [21].They concluded that the majority of them had a negative impact on QOL and psychosocial function. Thyroid-related Patient Reported Outcome questionnaire (ThyPRO) is yet another such questionnaire that evaluates thyroid patients’ QOL. The authors have recommended its use in clinical trials as it could additionally evaluate the relationships between clinical variables and QOL [22].

### Etiopathogenesis of TED

There are multiple mechanisms at work in TED, including autoimmune dysregulation, oxidative stress, hypermetabolic state, and vitamin D level imbalance. As mentioned earlier, the key underlying biology of this disease is the autoimmune-induced development of antibodies. Due to the dysregulation of the individual’s systemic thyroid status, there is infiltration of immune cells into the orbital tissue that activates the T and B cells, which produce antibodies to the TSH and the IGF-1 receptors present in the orbital fibroblast. Orbital fibroblasts appear to be the main target in TED, as they express both of these receptors. Antibody production against self-antigens (TSH R and IGF R) initiates the cascade of reactions that occur in TED. Autoantibody production is also noted in thyroid peroxidase (also known as antimicrosomal antibody) and thyroglobulin. Antibodies to the TSH receptor are also known as thyroid receptor antibodies (TRAb). TRAb is a heterogenous group of antibodies that can be thyroid-stimulating antibodies (TSAb), thyroid-blocking antibodies (TBAb), or neutral antibodies [23]. These immunoglobulins in turn activate the inflammatory cascade.

TSAb (TSH receptor-stimulating antibody) stimulates TSH-R, causing increased cyclic adenosine 3′,5′-monophosphate (cAMP), which increases the synthesis of thyroxine (T4) and triiodothyronine (T3). TBAb (a TSH receptor–blocking antibody) inhibits the function of the TSH-R and decreases the synthesis rate of thyroid hormones. Neutral Ab is able to induce variable signaling cascades, some of which are initiated by TSAb [24]. Both GD and HT are caused by lymphocytic infiltration in the thyroid parenchyma. In GD, since the infiltration is mild, the gland remains intact. In HD, the lymphocytic infiltrate causes the destruction of the follicles, which may lead to hypothyroidism [25].

The stimulation of these receptors by the antibodies can stimulate the inflammatory reaction, cause adipogenesis, and increase hyaluronan production [26]. TSH and IGF-1 receptors share common epitopes that are responsible for the physical and functional interactions in the fibroblast. Thus, inhibition of the IGF receptor can stop the progression of the cascading effects. Orbital fibroblasts in TED are different from normal orbital fibroblasts in terms of functionality and, hence, express abundant inflammatory cytokines [27].

The thyrotropin receptor (TSHR) was long thought to be the prime target in TED; however, recently, insulin-like growth factor-I receptor (IGF-IR) has been proposed as a second participating antigen in TED as it has physical and functional similarity, while inhibition of IGF-1R also causes signaling downstream from both receptors [28]. Teprotumumab, a human monoclonal antibody IGF-IR inhibitor, was used in moderate to severe active TED and was found to be safe and highly effective in reducing disease activity and severity. Induced cochlear deafness, however, was reported as a side effect. Targeting IGF-IR with specific biologic agents may result in a paradigm shift in the management of TED [29].

It has also been hypothesized that oxidative stress may cause the cascade of effects occurring in TED. Achieving a balance in the cell redox rate is recommended to maintain the normal homeostasis of cells. An increase in reactive oxygen species (ROS), such as hydroxyl radicals (OH^–^), hydrogen peroxide (H_2_O_2_), superoxide anions (O2), and lipid peroxides, and a decrease in their clearance can cause oxidative stress and the breakdown of cellular homeostasis. Glutathione (GSH), superoxide dismutase (SOD), glutathione peroxidase (GPX), and catalase act as ROS antagonists and maintain cellular redox homeostasis [30].

Dettore et al. hypothesized that the autoimmune insult causes DNA methylation which elicits differential gene expression, the latter being responsible for mediating the pathogenesis of TED. The authors added that 58 genes were differentially expressed in TED compared with the controls [27]. Thyrotoxicosis is a hypermetabolic state characterized by high consumption of intracellular ATP and oxygen. An increase in ROS can cause damage to mitochondria and affect the production of oxygen and ATP. In vitro studies in rats showed that exposure to H_2_O_2_ caused selenium (Se) deficiency and DNA damage, resulting in mutations and triggering apoptosis and necrosis [31]. It is hypothesized that the increase in ROS can cause dual actions, as follows: (a) an increased release of autoantigens and production of TSH-R autoantibodies (TRAb), and (b) orbital tissue damage that contributes to the clinical manifestations of hyperthyroidism.

There are a few atypical cases which stained positive for IgG4 association between TED and IgG4-related orbital disease. Both the entities have an overlap in the underlying autoimmune fibrous inflammatory disease possibly due to Β cell depletion [32].

### Role of genetics in TED

Genetic studies in TED have not been favored because of the small sample size of the previous studies and the inability to replicate the experiment. Moreover, the genetics of thyroid ophthalmopathy were mainly studied based on the genetic variants in GD and genes related to proinflammatory cytokines and immune regulation [33]. TED is a complex hereditary disorder which is, however, influenced by environmental factors; thus, it should be understood that the autoimmunity occurs when genetic susceptibility intersects with acquired factors. In the past two decades, common GD-associated genes identified and confirmed through linkage and association studies are HLA-DR1-Arg74 (human leukocytic antigen—DR beta 1 arginine at position 72), CTLA-4 (cytotoxic T lymphocytic antigen), PTPN22, CD40, CD25, TG (thyroglobulin), and the TSHR gene (thyroid-stimulating hormone receptor) [34].

Recently, Zou et al. used a data-driven approach to detect gene biomarkers in TED, which combines biomarkers both from those reported in the literature and those detected through their finding of differentially expressed genes in their study. Furthermore, a regulatory pathway of biomarkers was constructed, followed by various gene ontology-based enrichment analyses [35].

### Influence of epigenetics in TED

Epigenetic changes are modifications to DNA that cause genes to be turned on or off and thus influence the production of proteins in cells. They ensure that each cell produces only those proteins that are necessary for its function. These changes can be in the form of DNA methylation, histone modification, or non-coding RNA. In a Chinese study that compared the DNA methylation patterns between the healthy population and patients with TED, DNA methylation was significantly different between the groups in 148 genes. There are hyper- and hypomethylated genes, and the authors predict that they could be risk biomarkers for TED and could pave the way for new treatment strategies. In addition, decreased levels of ICAM1 methylation are significantly associated with exophthalmos in patients with GD [36].

### Risk factors in TED

Thyroid values are not the only risk factor, a large amount of literature having been published stating that TED is also influenced by deranged biochemical, hormonal, autoimmune, microbiome, and genetic factors. A multicenter cohort study concluded that free T3 and T4 are important predisposing risk factors for TED and that uncontrolled T3 and T4 play a critical role in the progression of the disease [37]. Female gender is a strong risk factor for TED; however, many studies report that men develop the most severe form of the disease. Middle-aged people are commonly affected, with the severity being greater in the elderly. White Caucasians are more commonly affected than Asians.

### Smoking and its association with TED

Smoking is an important risk factor for TED; however, it is not to date known which aggravates TED, smoke or cigare4e ingredients. The smoke (both active and passive) is believed to increase free radicals, causing oxidative damage that induces an inflammatory response leading to thyroid dysfunction [38]. According to the Tromso study, active current smoking is associated with a reduction in TSH levels in serum, which causes hyperthyroidism [39]. This was corroborated by The National Health And Nutrition Examination Survey (NHANES) study, which found significantly lower TSH values in active smoking patients (serum cotinine value of 15 ng/ml) compared to mild and occasional smokers [40]. The majority of studies have reported lower levels of TSH in patients with active smoking.

The effect of smoking on thyroid peroxidase is controversial and debatable. Thyroid peroxidase is an enzyme that adds iodine to thyroglobulin and is an important component of thyroid hormone synthesis [41]. Smoking has variable effects on thyroid function, which may be due to various constituents in tobacco, including carcinogens, alkaloids, and gases. Smoking has a protective effect on hypothyroidism and a negative influence on hyperthyroidism. Cigarette smoking is associated with increased orbital venous congestion in patients with TED [42].

### Diet and its association with TED

#### Relevance of iodine to TED

Iodine has a pivotal role in thyroid metabolism. Lack of iodine leads to many iodine-deficient disorders. This has been alleviated by the implementation of iodine fortification programs in many poor and underdeveloped countries around the world. On the other hand, excess intake of iodine is associated with immune dysregulation in susceptible individuals, which is associated with an increase in parenchymal destruction and apoptosis of the thyrocytes, causing hypothyroidism. According to one study, excess iodine intake is associated with an increase in thyroid peroxidase antibodies and thyroglobulin antibodies [43].

#### Relevance of vitamin D to TED

Vitamin D plays an important role in immunomodulation and its deficiency has been shown to cause various autoimmune diseases, including TED [44]. Vitamin D is obtained either through diet or sunlight and is responsible for the maintenance of the immune status. Vitamin D deficiency can induce immune dysregulation, generating autoantibodies in a vicious cycle. The immune system identifies the self-antigens as being foreign, that is, as non-self antigens. Due to immune intolerance, there is autoantibody production against the normal antigens of TSHR, thyroid peroxidase (also known as antimicrosomal antibody), and thyroglobulin [45]. Vitamin D supplementation could be an adjunct treatment strategy to slow disease progression due to its role in innate and adaptive immunity. There is an association between low levels of vitamin D and autoimmune disease.

#### Role of Se in TED

Se is essential for the deiodination process of thyroxine by deiodinases (DIOs). Deiodination is one of the vital steps in thyroid hormone synthesis. It is also needed for the degradation of excessive H2O2 generation (via glutathione peroxidases (GPXs) and thioredoxin reductase). A decrease in Se may increase the number of free radicals, resulting in oxidative damage. However, the beneficial effect of Se supplementation has not been proven. A double-blind, randomized controlled trial (RCT) of Se supplementation for 6 months in TED was associated with improved QOL, reduced soft tissue inflammation, improved appearance, and reduced progression of TED compared with placebo [46]. However, this study did not include measurements of Se concentrations in patients’ blood and was not confirmed by a second trial. Se plays an important role in thyroid metabolism. Changes in Se levels can affect thyroid function. Khong and Cols observed that Se serum values were lower in GD with TED suggesting that lower Se values are a strong risk factor [47]. Se is present in large quantities in the thyroid gland and is involved in thyroid hormone synthesis and metabolism.

#### Iron metabolism and its role in TED

Iron is required for thyroid synthesis, and there are many reports quoting a deficiency of iron in Hashimoto’s thyroiditis (HT). Iron is essential for bacterial growth, and iron availability influences the composition of the microbiota [48]. The common commensal bacteria, including *Lactobacillus* and *Bifidobacterium*, require less or no iron, but the pathogenic bacteria such as *Salmonella*, *Shigella*, and *E. coli* require iron for virulence and colonization. Humans live in a symbiotic relationship with the gut microflora [49]. There are trillions of bacteria in the gut with both good and pathogenic flora, which are responsible for the formation of gut-associated lymphoid tissue (GALT). They also play a major role in the absorption of various micronutrients, the synthesis of vitamins, the digestion of fibers, and the homeostasis of the immune system. An alteration in the composition of intestinal bacteria (dysbiosis) can cause an increase in intestinal permeability and give rise to an altered immune response. In addition, impaired microbiota (dysbiosis) influences antibody production against thyroid peroxidase and thyroglobulin, causing HT and hypothyroidism [50]. Dysbiosis can also stimulate autoantibody production to the thyroid-stimulating hormone (TSH) receptor, which results in hypersensitivity of the thyroid gland and hyperthyroidism [51].

#### Zinc metabolism and thyroid disorder

Zinc is an essential micronutrient required for thyroid function and homeostasis and is a catalyst for the conversion of T4 to T3. It also plays a role in gene expression. Zinc deficiency is a cause of subclinical hypothyroidism [52]. Moreover, since zinc is an important component of the antioxidant system, its deficiency can alter the immune system [53].

#### Role of cholesterol in TED

The role of cholesterol in TED should be considered in a dynamic manner since at the beginning of the disease, association with hyperthyroidism leads to low cholesterol levels, while during normalization of thyroid hormone levels, the basal lipid profile returns to the levels prior to the onset of GD.

Serum cholesterol levels show a strong association with TED and statins reduce the incidence of TED [54]. Furthermore, the hypolipemic effect of statins protects the eyes from developing TED. There is no significant difference in the development of TED in patients taking non-statin anticholesterol agents [55].

### Medications and TED

Medications causing abnormal thyroid function are amiodarone, lithium, interferon, tyrosine kinase inhibitors, rifampin, and anti-epileptics. Tyrosine kinase inhibitors are targeted therapies approved for the treatment of several hematological tumors [56]. There are various hypotheses explaining thyroid dysfunction, including destructive thyroiditis and inhibition of vascular endothelial growth factor (causing alteration of thyroid function). There are few case reports of hypothyroidism induced by rifampin [57].

Amiodarone is an anti-arrhythmic agent that affects the function of the thyroid gland because of its rich iodine content. Patients treated with amiodarone may manifest clinically significant amiodarone-induced hypothyroidism or amiodarone-induced thyrotoxicosis. Amiodarone-induced hypothyroidism is due to the inability of the thyroid to escape the Wolf-Chaikoff effect, and it prevails in areas with high iodine content. Amiodarone-induced hyperthyroidism occurs more frequently in areas with low iodine intake due to iodine-induced excessive thyroid hormone synthesis (type I) or destructive thyroiditis with release of preformed hormones (type II) [58].

### Current management of TED

#### Medical management

Patients with Graves’ ophthalmopathy should be managed by a team of endocrinologists and ophthalmologists with speciality experience in managing TED. Ophthalmic treatment must be tailored to the patient’s QoL, psycho-social effects, and severity and stage of disease. Management mainly depends on the activity of the disease.

Maintenance of euthyroid status is essential in all cases of TED. Antithyroid drugs (ATD) should be administered to patients with GD, while thyroid supplementation is given to hypothyroid patients. Among ATD, methimazole is the drug of choice. Propyl thiouracil is given to patients who are allergic to methimazole or who have thyroid storm or in pregnancy [59]. Chronic low-dose therapy with methimazole is reported to be a better alternative to radioactive iodine according to a recent review [60].

Alemtuzumab is a humanized monoclonal antibody targeting CD52 used for the treatment of multiple sclerosis, while there are few reports of TED with alemtuzumab therapy for GD [61]. A clinician should be aware of this and change therapy if there is clinical worsening.

Management of hyperthyroidism in women of childbearing age is presented in depth in a review by Ashkar et al. [62]. Contraception is advised until hyperthyroidism is controlled. ATD can be stopped in early pregnancy if thyroid levels are normal. The treatment is titered based on risk-benefit ratio.

Smoking cessation is mandatory in all phases because it worsens the outcome and is the only modifiable risk factor. For mild inactive disease, eye shades, artificial tears, elevation of head during sleep, and avoidance of eye cosmetics are suggested conservative treatments [63].

Under medical management, steroids are the main armamentarium when the disease is active. Radiation also reduces activity along with steroids, while immunomodulators help to modulate the disease and reduce the need for steroids.

#### Steroids

Based on current evidence and efficacy/safety profiles, costs and reimbursement, drug availability, and long-term effectiveness, EUGOGO guidelines recommend a combination of IV methylprednisolone and mycophenolate sodium as first-line treatment for moderate to severe active disease. IVMP is given at a dose of 0.5 g/week for 6 weeks along with 0.72 mg of mycophenolate sodium for 6 weeks. If there is response, taper IVMP to 0.25 gm weekly for 6 weeks and then mycophenolate sodium for 18 weeks. If the disease becomes inactive, then stop therapy and do cosmetic correction for proptosis or lid if the patient desires. If the disease is still active, go for second-line therapy with 0.75 gm/week for 6 weeks and then taper to 0.5 gm/week for 6 weeks. If the patient is non-responsive to steroids, then another second-line treatment should be tried [16].

Second-line treatments for moderate to severe and active TED include the following: (a) the second course of intravenous methylprednisolone (7.5 g) or (b) oral prednisone or prednisolone combined with either cyclosporine or azathioprine, or orbital radiotherapy combined with oral or IV glucocorticoids, or immunomodulators such as teprotumumab, rituximab and tocilizumab [16].

Sight-threatening TED is treated with several high single doses of intravenous methylprednisolone per week and, if the patient is unresponsive, is accompanied by urgent orbital decompression. National surveillance of dysthyroid optic neuropathy in the UK shows that 50% of patients with dysthyroid neuropathy end up with orbital decompression after initial steroid therapy [64].

A recent novel mode of steroid administration through nanotechnology was reported by Detiger et al. in Rotterdam. In this study, steroid-laden liposomes were injected into patients with active TED. The authors demonstrated the efficacy of this treatment in reducing activity in a subset of patients. The advantage of this targeted therapy is that lower doses are given at fewer hospital visits while avoiding the side effects of systemically administered steroid medication. Intraorbital steroid injection for resistant active TED cases has also been tried but remains controversial due to the potential side effects of steroid-induced intraocular pressure increase [65]. Dr. Goldberg calls this technique the ugly sister compared to oral steroids. He has shared his anecdotal experience, saying that intraorbital steroid injection in TED is very effective and safe when given employing the right technique [66].

#### Immunomodulators

With the accumulation of increasing knowledge about molecular and immunopathological mechanisms, the importance of immunomodulators has been stressed. Biological agents including rituximab, eternacept, adalimumab, desatinib, and intravenous immunoglobulin have been tried with good results according to some studies. These biological agents act in the early phase before inflammatory proteins are released [67].

Rituximab, a chimeric human and mouse monoclonal antibody against CD20 antigen on B cells, has been trialed for the treatment of active TED [68]. Rituximab was originally used to treat lymphoproliferative malignancies and is now being used in TED. It acts on B cells to prevent activation and differentiation through a variety of mechanisms. In a review published with cases treated with rituximab, the clinical activity was reduced; however, the authors stressed the need for long-term studies to establish its effectiveness [69]. Two prospective randomized clinical trials of patients with active moderate to severe TED have been conducted for rituximab, one in the USA and another in Italy, with conflicting results [70]. A Cochrane review has therefore concluded that there should be more multicenter studies to determine the efficacy of this drug [71].

Teprotumumab is a monoclonal antibody against IGF-1R and is the current FDA-approved drug for the treatment of active TED. Autoantibodies to the thyrotropin receptor (TSHR) resemble TSH, resulting in hyperthyroidism. Smith et al. reported that insulin-like growth factor I receptor (IGF-IR) is overexpressed in orbital fibroblasts (OFs) and forms a physical and functional complex with TSHR [72]. Its activity is necessary for mediation of components of downstream TSHR signaling; inhibition of IGF-IR activity with specific monoclonal antibodies can attenuate the induction of TSH. Two randomized controlled trials have been performed, one in the USA and the other in Europe. Both of these studies have shown a favorable response to teprotumumab in terms of reduction in proptosis and decrease in activity for TED [73].

The current recommendation for the use of teprotumumab is an intravenous dose every 3 weeks (10 mg/kg first dose, then 20 mg/kg) for a total of eight infusions. Common side effects include nausea, diarrhea, muscle spasms, hearing impairment, dysgeusia, headaches, dry skin, infusion reactions, alopecia, paresthesia, weight loss, and hyperglycemia [74].

Tumor necrosis factor (TNF) is a proinflammatory cytokine and is found to be elevated in TED serum. Paridaens et al. were the first to study the effect of anti-TNF (etanercept) in a case series of patients with moderate to severe active TED [75]. Later, adalimumab, an anti-TNF agent, was successfully used as a steroid sparing agent in inflammatory TED [76]. Infliximab infusion (an anti-TNF antibody) has been reported to reverse the activity of TED in 72 h [77].

Platelet-derived growth factor (PDGF) from orbital fibroblasts has been shown to be a key factor in the pathogenesis of TED: given that tyrosine kinase inhibitors target PDGF signaling, the authors report that these could be potential targets for therapeutic approaches [78]. Dasatinib, a thyrosine kinase inhibitor, reduces PDGF signaling and reduces orbital tissue expansion and inflammation. In a pilot study, this drug was found to be effective. The latter findings warrant future multicenter trials to learn more about the long-term outcomes. [78]

### Surgical management

Surgical management is generally required for cosmetic correction or functionally when there is dysthyroid optic neuropathy. The disease can cause disfiguring proptosis, lid retraction, or squint. Ideally, the order of surgery should be orbit, lid, and then squint. Proptosis is the most visible and obvious side effect of TED, which requires earlier correction. Correction of orbital proptosis itself could improve lid and squint and can then be adjusted if revisions are needed [79]. Orbital decompression involves removal of one or multiple orbital walls with or without orbital fat removal to increase orbital volume and reduce the pressure exerted by the expanded extraocular muscles on the optic nerve. Indications for this surgery include compressive optic neuropathy, disfiguring proptosis, exposure keratopathy, globe subluxation, and/or uncontrollable elevation of intraocular pressure. The patient should be euthyroid when cosmetic corrections are undertaken.

### Biomarkers

Biomarkers can help in prediction of the disease. They can also be targets for treatment, such as the IGFR receptor antibody (teprotumumab), which is currently being used to treat active TED. With the advent of newer molecular target therapies, earlier treatment can be initiated for TED even before the activity develops. This could in turn avoid the complications with current treatment protocol.

As the biogenesis of the disease is an inflammatory cascade, proinflammatory cytokines and chemokines including IL-1, IL-6, IL-10, IL-8, C–C chemokine ligand 20 (CCL20), and IL-17 are elevated. Studies have shown that these markers can also indicate the stage of activity of the disease and report that the blood levels of some cytokines reflect the response to treatment: patients with refractory TED have higher levels of IL-4, IL-6, and IL-10 than patients in remission. Intercellular adhesion molecule-1 (ICAM-1) and soluble vascular cell adhesion molecule-1 (sVCAM-1) have been found to be elevated in the blood of TED patients as compared to controls [35].

## Conclusion

TED is a real challenge for both the patient and the clinician, exerting a considerable psychosocial and financial impact on affected individuals. It is self-limiting with active and inactive phases. The current management of the disease focuses mainly on controlling symptoms. Early diagnosis and timely management are essential, while reduction of certain risk factors such as smoking and cholesterol levels are likely to help patients in the long run. Biomarker evaluation as a source of diagnostic and prognosticative markers is evolving, which could aid in gaining a better understanding of the pathophysiology of the disease and enable new personalized therapeutic strategies. They may also be used in future guidelines or standard protocols on disease-modifying agents.
